# Decoding and reconstructing yeast protein flavor based on an integrated sensory-omics approach

**DOI:** 10.1016/j.fochx.2026.104060

**Published:** 2026-06-09

**Authors:** Jiahui Chen, Boya Cao, Zikang Xu, Baoguo Sun, Shihao Sun, Dandan Pu, Lili Zhang, Yuyu Zhang

**Affiliations:** a Key Laboratory of Geriatric Nutrition and Health (Beijing Technology and Business University), Ministry of Education, 100048, China; b Key Laboratory of Flavor Science of China General Chamber of Commerce, Beijing Technology and Business University, 100048, China; c Food Laboratory of Zhongyuan, Beijing Technology and Business University, 100048, China; d Beijing Life Science Academy, Beijing 102209, China

**Keywords:** Yeast proteins, Key aroma compounds, GC × GC-TOF-MS, Recombination experiment, Omission test

## Abstract

To enhance the sensory quality of yeast proteins and facilitate their broader application in food products, its key aroma compounds were systematically characterized through sensory evaluation and GC × GC-TOF-MS. The combined use of three extraction techniques (SAFE, SDE, and SPE) enabled the identification of 104 odor-active compounds. Sensory profiling revealed the roasted, sour, sweet, and almond-like were dominant. Among the three extraction methods, SAFE and SDE were optimal for bulk recovery, while SPE targeted organic acids. The aroma recombination experiments confirmed the recombination model based on the three methods demonstrates a relatively high similarity (90%) with the protein sample. The omission experiments further confirmed that benzaldehyde, *n*-decanoic acid, hexanoic acid, methyl benzoate, geranylacetone, 1-nonanol, 2-decanol, 2-pentadecanone, farnesol, *γ*-undecalactone, and octanal were identified as key aroma compounds in yeast proteins. The work establishes a robust analytical framework for yeast protein flavor characterization, aiding product development and quality control.

## Highlights

• A total of 104 aroma-active compounds were characterized in yeast protein (YP).
• SAFE and SDE were more effective for bulk recovery, accounting for over 80% of the extracted YP mass.
• Eleven key aroma compounds were found to contribute greatly to the overall aroma of the YP samples.
• GC × GC-TOF-MS revealed three odorants that escaped GC–MS detection yet were sensorially perceptible.

## 1 Introduction

The continuous growth of the global population and the increasing demand for sustainable protein resources have driven the exploration of alternative protein sources (Guillemot et al., 2004). The growing population size will inevitably have an impact on the demand for food, especially protein. Protein is an essential macronutrient that plays a critical role in daily nutrition. Its importance spans multiple physiological functions, including muscle building and maintenance, immune regulation, bone health, metabolic control, and energy provision (Ajomiwe et al., 2024). Yeast protein is a high quality microbial protein extracted from low-nucleic acid yeast through bioengineering technology, with the nutritional characteristics (Ma et al., 2023) of high protein and low fat. Yeast protein is generally regarded as a high-quality protein source, rich in all essential amino acids. Its amino acid profile is comparable to that of certain plant proteins, and similar to that of animal proteins. In addition, yeast protein typically exhibits high digestibility, allowing for effective absorption and utilization by the human body, thereby supporting muscle growth, repair, and other physiological functions. Moreover, yeast has a short growth cycle and is widely available as a raw material, making it a cost-effective and sustainable protein source with high production efficiency and a low environmental footprint (Qiao et al., 2025). Its production process adopts enzymatic technology to decompose the yeast cell wall, followed by gradient centrifugation and high-pressure homogenization to achieve the dispersion and enrichment of the active ingredients, and then purification and refinement through multi-stage membrane separation technology, and finally through instantaneous sterilization and spray-drying to ensure that the product meets the food safety standards (Chen et al., 2019). Currently, brewer's yeast protein has been approved in the United States as a nutritional supplement to be added to food, and the European Union has also approved it as a new food ingredient, neither of which has a limit on the amount of consumption. In December 2023, the yeast protein was included in the catalog of new food ingredients by China's National Health Commission with only a restriction on the applicable population (Podpora et al., 2015). However, the yeasty odor present in yeast proteins directly affects product palatability (Wang et al., 2020; Zheng et al., 2020), severely limiting the development and promotion of yeast protein-based products. To date, the key aroma compounds of yeast protein remain poorly understood. Off-flavor formation in yeast protein is not an inherent property; instead, it is strongly influenced by several factors, including yeast strain, cultivation conditions (Vrzal et al., 2023), processing methods, and degradation reactions that occur during storage.

Sensoryomics is a molecular-level analytical method that identifies key flavor compounds in food by establishing links between volatile compounds and sensory perception, thereby elucidating the sensory and flavor profiles of food (Han et al., 2026). Vrzal et al. (2023) employed a sensoryomics approach to comprehensively characterize lager beers fermented by four traditional yeast strains. By comparing the chemical and sensory profiles of the beers, they demonstrated the theoretical importance of sensory interactions in beer flavor perception. Selecting the appropriate extraction methods for analyzing volatile compounds in yeast proteins is of an important step in aroma analysis. Conventional extraction techniques, including solvent-based extraction, steam distillation, adsorption-desorption processes, headspace sampling, and supercritical fluid extraction have been extensively employed in previous studies (Zhang et al., 2023). SDE operates by co-heating the aqueous sample phase and immiscible organic solvent to boiling temperatures. While this method consistently demonstrates superior recovery rates for most compounds, the inherent thermal exposure renders it incompatible with thermosensitive substances (Chaintreau, 2001). Lin et al. (2014) employed dynamic headspace extraction (DHE) and SDE to extract aroma-active compounds from yeast extracts. Their results demonstrated that SDE recovered a higher number of key flavor compounds from yeast extracts, exhibiting significantly greater sensitivity toward sulfur-containing compounds and pyrazines compared to DHE. SAFE is a technique for extracting volatile compounds from samples under mild thermal conditions (40–50 °C) and high vacuum. This combination effectively preserves the original chemical profile by minimizing thermal degradation and preventing the formation of degradation products during the extraction process, thereby achieving high recovery rates of target analytes (Louw, 2020). Zhang et al. (2017) investigated flavor compounds in yeast extracts using solid-phase microextraction (SPME) and SAFE methods. A total of 30 aroma-active compounds were identified, with 4-methylphenol, 3-methylpyridine, 3-methylbutyric acid, and propionic acid characterized as primary contributors to the characteristic off-flavor in the yeast extracts. SPE is a chromatographic technique that enables selective elution of volatile compounds through solvent-mediated desorption after initial adsorption on an SPE solid phase extraction columns. Although this approach demonstrates advantages in solvent economy and operational efficiency compared to traditional liquid-liquid extraction, systematic method development involving preliminary experiments is required to optimize critical parameters (e.g., sorbent selection, solvent polarity, and elution volume) for achieving satisfactory recovery rates (Xu et al., 2007). The optimized SPE method delivered very low detection and quantification limits for the analytes, demonstrating high sensitivity (Yang et al., 2011). Thus, integrating multiple extraction methods is crucial for the precise identification of key odor-active compounds derived from yeast proteins. SAFE is performed under mild conditions (low temperature, high vacuum) and causes minimal thermal degradation or artifact formation. It is widely regarded as the most reliable method for obtaining a near-native volatile profile of food matrices, especially for heat-labile compounds. SPE is also carried out at ambient temperature and involves no prolonged heating. It is effective for retaining a broad range of volatile compounds, particularly those with moderate to low polarity. This method offers high extraction efficiency for trace volatile compounds in samples and significantly improves the detection of high-molecular-weight compounds. Therefore, the three techniques complement each other, together providing a more comprehensive profile of the odor-active compounds.

The Comprehensive Two-Dimensional Gas Chromatography-Time-of-Flight Mass Spectrometry (GC × GC-TOF-MS) system utilizes orthogonal separation through two serially coupled chromatographic columns with distinct selectivity mechanisms. This dual-column configuration, combined with high-resolution time-of-flight mass spectrometric detection, provides enhanced peak capacity and sensitivity, enabling comprehensive characterization of complex mixtures such as environmental samples or biological matrices (Chen et al., 2024; Shen et al., 2023). This hyphenated technique (GC × GC-TOFMS) has been widely used for the identification of unknown volatile compounds in complex samples, including food products (e.g., baijiu, fruit) (Szymczak et al., 2024; Yang et al., 2021).

Given that single extraction method cannot simultaneously preserve all aroma-active compounds due to differences in solubility and thermal instability of yeast volatiles, a combined approach employing SDE, SAFE, and SPE was adopted in this study. The objectives of this study were to (1) systematically compare the performance of three extraction methods (SPE, SAFE, and SDE) in isolating volatile compounds from yeast proteins; (2) characterize odor-active compounds through comprehensive analytical approaches combining GC-Olfactometry, GC–MS, and GC × GC-TOF-MS with subsequent quantification via external standard curves and odor activity value (OAV) calculations; (3) reconstruct the flavor profile of yeast protein by recombination and omission experiments based on the identified key odorants, thereby validating their actual contribution to the overall aroma. This study contributes to a better understanding of yeast protein flavor, providing data support for the development of yeast protein-derived products and the targeted enhancement of their sensory qualities.

## 2 Materials and methods

### 2.1 Materials

The yeast protein (YP) used in the experiment were provided by Angel Yeast Co., Ltd. (Hubei, China). The yeast proteins used in this study were derived from *Saccharomyces cerevisiae*. Yeast protein is produced via liquid deep-culture fermentation. After fermentation, the yeast cells are collected by centrifugation, then inactivated and washed to remove nucleic acids. A second centrifugation step yields a yeast slurry. The cell walls are subsequently removed by enzymatic hydrolysis, and the product is further refined through extraction, separation, purification, and drying.

### 2.2 Chemicals

Dichloromethane (≥99.9%, HPLC grade), 1,2-dichlorobenzene (≥99.9%, HPLC grade). Serial n-alkanes (C7 ∼ C40) with a purity of 99.9% were obtained from Sigma-Aldrich (Shanghai, China). Dimethyl trisulfide, 3-ethyl-2,5-dimethyl-pyrazine, 4-methyl-pyrimidine, 2-ethyl-6-methyl-pyrazine, methyl benzoate, 2-nonanone, octanal, naphthalene, γ-dodecalactone, nonanal, 2-decanone, dimethyl disulfide, heptanal, ethylbenzene, propanoic acid, salicylaldehyde, hexanal, 2-octanone, acetophenone, γ-undecalactone, 1-octen-3-ol, nerolidol, 6-methyl-5-hepten-2-one, 2,4-di-tert-butylphenol, indole, benzothiazole, 3,7,11-trimethyl-2,6,10-dodecatrien-1-ol, cis-3-hexenyl benzoate, phenol, 2-pentadecanone, γ-decalactone, 2,6-dimethylpyrazine, octanoic acid, 2-decanol, 2-furanmethanol, *o*-cresol, 1-nonanol, phenylethyl alcohol, nonanal, geranylacetone, benzyl alcohol, benzaldehyde, 2-piperidinone, pentanoic acid, dodecanoic acid, ethyl butyrate, dodecanoic acid, 3,7,11-trimethyl-2,6,10-dodecatrien-1-ol, alpha-bisabolol, hexanoic acid, *n*-decanoic acid, 2,5-dimethyl-benzaldehyde, α-methyl-benzenemethanol, and heptanoic acid with purity of ≥98% were purchased from Macklin Technology Co., Ltd. (Shanghai, China).

### 2.3 Sensory evaluations

Sensory evaluations were performed in clean and quiet room at 25 ± 1 °C. The twelve trained panelists with sensory evaluation experiences (6 female, 6 male, age range 24–49 years) were recruited from the College of Food and Health at Beijing Technology and Business University. Panelists were trained by standardized reference solutions representing seven aroma attributes: fatty (*E*,*E*)-2,4-decadienal), roasted (2,6-dimethylpyrazine), sour (propanoic acid), sweaty (butanoic acid), green (nonanal), sweety (benzyl alcohol) and almond-like (benzaldehyde). In accordance with ISO 5496:2006, an intensity score of 1 point was assigned to the detection threshold concentration of these compounds. Scores of 2 to 9 points were then assigned to concentrations corresponding to 2 to 9 times the detection threshold, respectively. Odor intensity was quantified using a 9-point scale: 1–3 (weak perception); 4–6 (medium intensity); 7–9 (strong intensity). The trained panelists were informed the experimental contents and detailed procedures, and they agreed to sign the informed consent form. The sensory evaluation test was approved by the ethics committee of Beijing Technology and Business University in 2024 (BTBU2024177).

### 2.4 Solvent-assisted flavor evaporation (SAFE)

The YP (20.00 g) and the dichloromethane (100 mL) were combined in a separatory funnel. Immediately add the internal standard solution (10 μL) (containing 0.05 mg/mL 2-octanol, 0.054 mg/mL 1,2-dichlorobenzene, and 0.046 mg/mL 2-methyl-3-heptanone in methanol). Then the mixture was stirred with CYLDZ-6 separation funnel vertical shaker (Beijing GHGK Automated Technology Academy, Beijing, China) at 250 rpm for 30 min to facilitate dissolution of volatile compounds into the dichloromethane phase. At the end of the shaking, the mixture was filtered and the organic phase (upper layer) was collected. The extraction was repeated in three times. The aroma compounds from organic phases were then isolated by SAFE device at the high vacum. The extraction parameters (vacuum: 1 × 10^−5^ Pa to 1 × 10^−6^ Pa; circulating water bath and distillation flask temperature: 40 ± 1 °C) were the same as our previous work (Pu et al., 2022). In the SAFE process, the solvent extract, remaining as a thin film or droplets in the separation flask, was gently vaporized. The volatiles were then cryo-trapped (condensed and frozen) in the collection flask using liquid nitrogen. Finally, the samples were concentrated to 3–4 mL by rotary evaporator and then concentrated to 1 mL by nitrogen blower. The YP concentrate was immediately analyzed by GC–MS and GC-O/AEDA. All analyses were performed in triplicate.

### 2.5 Simultaneous distillation extraction (SDE)

An improved version of the Likens-Nickerson-type simultaneous distillation extraction (SDE) apparatus was used to extract the volatile compounds from YP. The YP (50.00 g) and deionized water (300 mL) were loaded into a round-bottom flask (1 L). Immediately, the 70 μL internal standard solution (0.05 mg/mL 2-octanol, 0.05 mg/mL 1,2-dichlorobenzene, and 0.05 mg/mL 2-methyl-3-heptanone in methanol) was added. The reflux apparatus was assembled, with the extraction flask charged with dichloromethane (60 mL) was immersed in a 60 °C water bath, while the reaction flask (sample) was heated by oil bath at 130 °C. The extraction was maintained for 2 h under reflux conditions after both the sample and solvent phase reached their respective boiling points. The YP extract was dehydrated by adding 5.00 g of anhydrous sodium sulfate. Finally, the YP extract were concentrated to 1 mL by rotary evaporator and nitrogen blower. The rotary evaporation process was carried out at 25 °C (water bath temperature). The concentrate was stored in a − 40 °C for further analysis. All analyses were performed in triplicate.

### 2.6 Solid phase extraction (SPE)

The YP (5.00 g) mixed with of pure water (30 mL) were loaded into Separatory funnel (100 mL). The 70 μL internal standard solution (0.05 mg/mL 2-octanol, 0.05 mg/mL 1,2-dichlorobenzene, and 0.05 mg/mL 2-methyl-3-heptanone in methanol) was added for quantification. Then the mixture was stirred with CYLDZ-6 separation funnel vertical shaker at 250 rpm for 30 min to facilitate dissolution of volatile compounds into aqueous phase. After the mixture was filtered the supernatant was collected. The volatile compounds in YP were enriched by SPE with a LiChrolut EN solid-phase extraction (SPE) cartridges (200 mg, 3 mL, Merck, Darmstadt, Germany) (Pu et al., 2020). The material is a styrene/divinylbenzene copolymer and is commonly used for the analysis of aromatic and phenolic compounds in aqueous sample matrices. First, the LiChrolut EN cartridge was activated using dichloromethane, methanol and water. Then, the supernatant was slowly dripped into the LiChrolut EN cartridge. Sequentially the column was eluted with 4 mL aliquots of hexane/dichloromethane mixtures in varying volume ratios (0:4, 1:3, 2:2, 3:1, 4:0) until no detectable odor was observed in the eluate. Finally, the eluted fraction was concentrated to 500 μL by nitrogen blowing for further analysis. All analyses were performed in triplicate (*n* = 3).

### 2.7 Gas chromatography-olfactometry-mass spectrometry (GC–MS/O) and gas chromatography-mass spectrometry (GC–MS)

An Agilent 8890 GC system (Agilent Technologies, Santa Clara, CA, USA) coupled with a 5977B MSD and olfactometer (ODP3, Gerstel, Germany) was employed for the separation and identification of volatile compounds. The DB-WAX column (30 m × 0.25 mm i.d., 0.25 μm film thickness, Agilent Technologies, Santa Clara, CA, USA) was used for the separation of volatiles. The analysis was performed in electron ionization (EI) mode with a mass scan range of *m/z* 40–500. The aroma isolation and detection parameters of GC–MS analysis were reference from our previous study (Chen et al., 2023). Ultrapure helium (99.999%) was employed as the carrier gas with a constant flow rate of 1.00 mL/min in splitless injection mode. However, GC–MS/O analysis was performed using split injection (1:1) to optimize odorant perception at the sniffing port. All other chromatographic parameters were identical.The temperature program for the column oven was set as follows: initial temperature was held at 35 °C for 1 min, and linearly increased to 100 °C at 4 °C/min for 1 min, then increased to 170 °C at 2 °C/min for 1 min, and finally increased to 220 °C at 5 °C/min and held on for 1 min.

### 2.8 GC×GC-TOF-MS

The analytes was performed using an Agilent 8890GC (Agilent Technologies, Santa Clara, CA, USA) equipped with a J&X SSM1810 cryogenic jet modulator (J&X Technologies, Shenzhen, China) for two-dimensional separation and an Agilent 7250 A time-of-flight mass spectrometer (TOF-MS) detector. A DB-5 gas chromatographic column (60 m × 0.25 mm, 0.25 μm film thickness; Agilent Technologies, Santa Clara, CA, USA) and a DB-17 gas chromatographic column (0.85 m × 0.25 mm, 0.15 μm film thickness; Agilent Technologies) were employed as the first- and second-dimensional columns, respectively, for volatile compounds separation in comprehensive two-dimensional gas chromatography (GC × GC) analysis. High-purity helium (99.999%) was employed as the carrier gas with a constant flow rate of 1.3 mL/min in splitless injection mode. The chromatographic separation utilized a temperature program initiated at 50 °C (hold time: 0.5 min), followed by a linear ramp of 4.5 °C/min to 320 °C, and finally maintained at 320 °C for 5 min. Effluents from the dual capillary columns were introduced into the ionization chamber operating at 70 eV electron impact energy. Mass spectra were acquired in full-scan mode across the *m/z* range of 30–650, with instrumental parameters optimized as follows: ion source temperature was 230 °C, transfer line temperature was 250 °C. To enhance chromatographic resolution in comprehensive two-dimensional separation, the cryogenic modulator was programmed. The temperature in the cold phase was set to - 51 °C with a modulation time of 4 s (Huo et al., 2021; Qian et al., 2022; Xu et al., 2022).

### 2.9 Aroma extraction dilution analysis (AEDA)

GC–MS/O combined with aroma extract dilution analysis (AEDA) was employed to evaluate the aroma-active compounds from the three YP aroma extract samples. Each extract was serially diluted with dichloromethane using a 2-fold dilution series (1,2, 1:4, 1:8, …, up to 1:1024). One microliter of each dilution samples were injected into the GC system and analyzed by GC–MS. Three trained sensory panelist simultaneously evaluated the GC effluent until no odor was detected. The flavor dilution (FD) factor was defined as the highest dilution at which the odorant could still be detected by the evaluator (Li et al., 2023).

### 2.10 Qualification and quantification analysis

The identification of aroma compounds in YP was systematically conducted through multiple analytical approaches, including mass spectral library matching, retention index (RI) verification, comparison with authentic standards, and odor attributes. For mass spectral analysis, compound identification was performed using the NIST 20.0 reference database with rigorous matching criteria. Retention indices were determined through a series of n-alkanes (C_7_-C_40_) analyzed under same chromatographic conditions to ensure analytical consistency (Zhu et al., 2016). Quantitative determination employed an internal standard calibration methodology. The internal standard calibration curve method was used to quantify odor-active compounds with high FD factors (FD ≥ 2) (Zhang et al., 2018). Standard compounds were dissolved in dichloromethane to prepare a stock solution. The stock solution was grouped and mixed according to the retention times, qualifying and quantifying ions, and concentrations of the compounds. The mixture was then serially diluted to obtain seven concentration levels. Subsequently, 2-methyl-3-heptanone (IS1), 1,2-dichlorobenzene (IS2), and 2-octanol (IS3) were added at concentrations matching those in the samples to serve as internal standards (IS). Finally, a calibration equation was constructed by plotting the ratio of peak areas against the corresponding concentration ratios (the ratio of the analyte to the internal standard). Quantitative analysis was performed in selective ion monitoring (SIM) mode based on the qualifying and quantifying ions of each compound, with a dwell time of 200 ms.

For each analyte, a calibration curve was constructed by plotting the peak area ratio (*A*/*A*_*IS*_) of the analyte to the corresponding internal standard against the concentration ratio (*C*/*C*_*IS*_). The calibration equation was fitted by linear regression:

(1) $$\frac{A}{A_{\mathrm{IS}}}=a\times \frac{C}{C_{\mathrm{IS}}}+b$$

where a is the slope and b is the intercept. The concentration of each analyte in the sample was then calculated by interpolating its measured peak area ratio onto the corresponding calibration curve. This method corrects for both injection volume variations and matrix effects, ensuring accurate quantification.

### 2.11 Odor activity value (OAV)

OAV ratio of concentration to the corresponding aroma threshold value, is the key to identify the contributions of aroma-active compounds to the characteristics aroma of YP. Compounds with OAV >1 were the potential aroma contributors. The thresholds were determined using the S-curve fitting method. Specifically, a series of concentration gradients (typically 7 levels spanning three orders of magnitude) were prepared for each compound. Panelists (*n* = 17) were asked to indicate whether they perceived the odor in a three-alternative forced-choice (3-AFC) paradigm. The proportion of correct responses was plotted against the logarithm of concentration, and a sigmoidal (S-shaped) curve was fitted. The threshold concentration (e.g., 50% correct rate, often referred to as the best-estimate threshold, BET) was interpolated from the fitted curve. This method is widely used in sensory analysis and follows the principles described in ISO 13301.

### 2.12 Recombination and omission experiments

To validate the applicability of the flavor reconstruction model, panelists evaluated the reconstructed samples against yeast protein samples via an attribute-by-attribute comparison. The odorless yeast protein matrix was prepared through sequential solvent extraction by dichloromethane, methanol, purified water, and ethanol, with each solvent undergoing three cycles of 30-min washing, followed by drying to completely remove residual solvent. The resulting matrix was confirmed to be odorless by sensory evaluation. Subsequently, the aroma recombination process involved incorporating odor-active compounds with OAV > 10 (identified through dual extraction methods) into an odorless ultrapure solution. A trained sensory panel conducted comparative evaluations of both the native yeast protein samples and the recombination models to assess their sensory characteristics.

Each omission model was evaluated against the recombination reference model using triangular discrimination tests (Xiao et al., 2017). The models were assigned randomized numerical codes to eliminate bias, and trained sensory panelists were tasked with two objectives: (1) identifying whether a sample could be sensorially distinguished from the reference and (2) characterizing its predominant sensory attributes through descriptive analysis for those samples exhibiting discernible differences.

### 2.13 Statistical analysis

The sensory data was analyzed using one-way analysis of variance (Duncan test) with SPSS 17.0 software (SPSS Inc., Chicago, IL). The sensory profile of YP was created using Origin 2025 software (OriginLab Corporation). Results are expressed as the means ± standard deviations from three measurements. To determine whether the omission of a specific compound led to a perceivable sensory difference, triangle tests were conducted according to ISO 13301. For *n* = 12 subjects, the minimum number of correct judgments required to determine significance at the α = 0.05 level was 8. If the number of correct judgments is ≥8, this indicates a statistically significant difference (*p* < 0.05), confirming that the absence of the target compound has a detectable effect on sensory perception. If the number of correct judgments reaches ≥9, the difference is considered highly significant (*p* < 0.01). Otherwise, results with ≤7 correct judgments are considered non-significant (*p* ≥ 0.05).

## 3 Results and discussion

### 3.1 Complementarity between SAFE, SPE and SDE

**Fig. 1:**
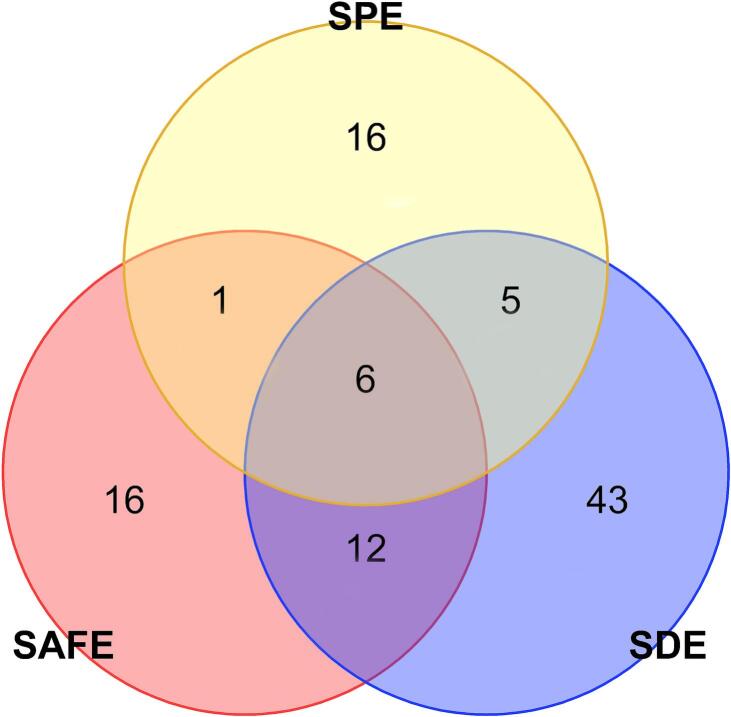
Comparison of aromas using three analytical methods.

A comprehensive aroma analysis of YP extracts revealed 104 aroma-active compounds, of which 99 were identified. The Venn diagram includes only the 99 identified compounds; the 5 unidentified compounds were excluded. A total of 83 volatile compounds were effectively isolated and characterized through complementary application of SAFE and SDE techniques, as demonstrated in Fig. 1. Quantitative analysis revealed that 85% of flavor compounds present in YP matrices were successfully recovered through the combined use of these two extraction methods, indicating their synergistic potential for comprehensive aroma profiling. This methodological combination establishes an optimized framework for YP flavor substance extraction, serving as a valuable reference for food matrix analyses. Notably, SPE demonstrated unique advantages in identifying high-abundance organic acids with OAV > 1, which were found to make substantial contributions to the overall flavor profile of YP.

### 3.2 Analysis of volatile compounds obtained by GC×GC-TOF-MS

**Fig. 2:**
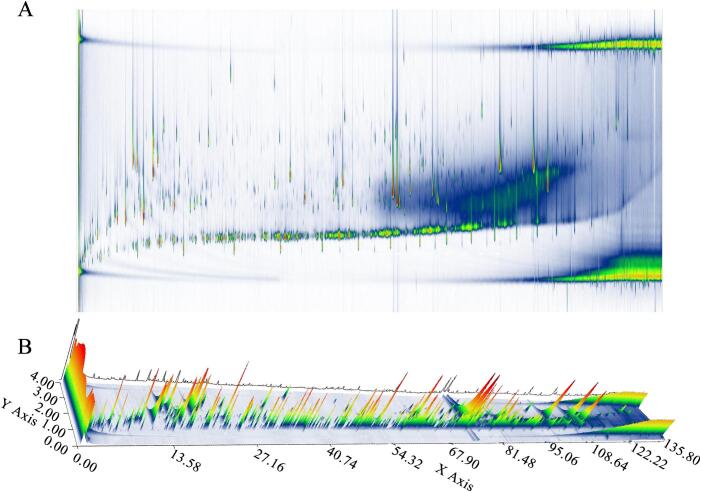
Full two-dimensional chromatographic profile and three-dimensional diagram of yeast protein samples (simultaneously distilled and extracted).

The SDE aroma extract of yeast protein (YP) was analyzed by GC × GC-TOF-MS; the other extracts (SAFE and solvent extraction) were analyzed by conventional GC–MS and GC-O. The total ion chromatogram (TIC) of volatile compounds extracted from yeast protein via SDE is shown in Fig. 2A. The horizontal axis corresponds to the first-dimension retention time, and the vertical axis represents the second-dimension retention time. Signal intensity in the TIC is color-coded, with blue to red indicating progressively higher relative abundances. A three-dimensional representation of the same data is provided in Fig. 2B. After eliminating ineffective peaks such as column bleeding, compound identification was conducted through spectral matching against the NIST20 library. The analytical method employed a forward column configuration: a non-polar primary column coupled with a mid-polarity secondary column. Consequently, components with higher polarity exhibit longer retention times on the secondary column, resulting in a spatial distribution where compounds are arranged from bottom to top in order of increasing polarity. Based on the two-dimensional distribution patterns of the sample (manifested as the tiling effect among homologous compounds) and the characteristic mass spectral ions, the spatial distribution of different compound families within the chromatographic plane was determined. Using n-alkane standards from C_7_ to C_40_ for retention index (RI) calibration, along with NIST20 library matching, qualitative identification of various compound classes was achieved. Retention indices obtained on the DB-WAX column for volatile compounds detected by comprehensive GC × GC-TOF-MS (Table S1) were compared with those from GC-O (Table 1). This comparative approach allowed identification of odor-active compounds that were not detected by GC–MS but were perceptible by olfactory evaluation.

**Table 1:** Identification of the aroma-active compounds in YP.

No.	Aroma-active compounds	CAS	Aroma quality	Isolations	RI	FD	Identifications
1	Pentanal	110-62-3	Almond, bitter, malt	SDE	984	–	MS/O
2	Diacetyl	431-03-8	Butter, pastry, yeast	SDE	998	–	MS/O
3	2-Methyl-3-buten-2-ol	115-18-4	Herbal, earthy	SDE	1018	–	RI/MS/S/O
4	Ethyl isovalerate	108-64-5	Fruity apple, buttery, banana, blueberry	SDE	1070	–	RI/MS/S/O
5	Disulfide dimethyl	624-92-0	Cabbage, garlic, onion	SDE	1070	4	RI/MS/S/O
6	2-Hexanone	591-78-6	Fruity, fungal, buttery	SDE	1075	–	RI/MS/S/O
7	Hexanal	66-25-1	Fatty, green, grassy	SDE	1099	8	RI/MS/S/O
8	Ethylbenzene	100-41-4	Fragrant	SDE, SPE	1120	4	RI/MS/S/O
9	1-Butanol	71-36-3	Winey, fusel oil-like	SDE	1134	4	RI/MS/S/O
10	4-Methyl-2-pentanol	108-11-2	Pungent	SDE	1157	–	RI/MS/O
11	1,3,5-Trioxane	110-88-3	Formaldehyde odor	SDE	1163	–	RI/MS/S/O
12	2-Heptanone	110-43-0	Fruity, cinnamon	SDE	1175	–	RI/MS/S/O
13	Heptanal	111-71-7	Fatty, pungent	SDE	1178	–	RI/MS/S/O
14	1-Pentanol	71-41-0	Alcoholic-breathtaking, fusel-like odor	SDE	1239	–	RI/MS/S/O
15	Unknown1		Yeasty	SAFE	1241	2	O
16	Styrene	100-42-5	Sweet, balsamic, almost floral	SDE	1244	–	RI/MS/S/O
17	2-Methylpyrazine	109-08-0	Nutty, cocoa, roasted, chocolate	SDE	1255	–	RI/MS/S/O
18	Octanal	124-13-0	Fatty, citrus	SAFE, SDE	1270	64	RI/MS/S/O
19	2-Octanone	111-13-7	Floral, green, fruity	SAFE, SDE	1283	32	RI/MS/S/O
20	2,6-Dimethyl-pyrazine	108-50-9	Chocolate, roasted nuts	SAFE, SDE	1315	256	RI/MS/S/O
21	*N*,*N*-Dimethylformamide	68-12-2	Fishy, ammonia	SAFE	1319	–	RI/MS/S/O
22	4-Methyl-pyrimidine^a^	3438-46-8	Roasted, steamy aroma	SDE	1324	4	RI/MS/S/O
23	1-Hexanol	111-27-3	Fragrant, mild, sweet	SDE	1342	–	RI/MS/S/O
24	6-Methyl-5-hepten-2-one	110-93-0	Fatty, green, citrus-like	SAFE	1365	128	RI/MS/S/O
25	2-Nonanone	821-55-6	Rose and tea-like	SAFE, SDE	1387	16	RI/MS/S/O
26	Nonanal	124-19-6	Fatty, citrus-like	SAFE, SDE	1388	16	RI/MS/S/O
27	Unknown2		Floral	SAFE	1408	32	O
28	3-Ethyl-2,5-dimethyl-pyrazine^a^	13360-65-1	Baked potato fragrance	SDE	1432	512	RI/MS/S/O
29	3-Furaldehyde	498-60-2	Cocoa, roasted	SDE	1444	–	RI/MS/S/O
30	1-Octen-3-ol	3391-86-4	Mushroom	SAFE, SDE	1450	32	RI/MS/S/O
31	Linalool oxide	34995-77-2	Floral	SDE	1454	–	RI/MS/S/O
32	2-Ethyl-1-hexanol	104-76-7	Rose and sweet, fatty-floral	SDE	1478	–	RI/MS/S/O
33	2-Decanone	693-54-9	Orange-like, cheese fatty, dairy creamy	SDE	1478	4	RI/MS/S/O
34	Decanal	112-31-2	Sweet, green, orange peel, citrus	SAFE	1494	–	RI/MS/S/O
35	Benzaldehyde	100-52-7	Bitter almond	SAFE, SDE, SPE	1503	128	RI/MS/S/O
36	Propanoic acid	79-09-4	Rancid	SPE	1508	8	RI/MS/S/O
37	4-Cyclopentene-1,3-dione	930-60-9	Herbal	SDE	1517	4	RI/MS/S/O
38	1-Octanol	111-87-5	Orange-rose odor, sweet, herbaceous	SDE	1546	–	RI/MS/S/O
39	Linalool	78-70-6	Camphoraceous and terpenic notes	SDE	1551	–	RI/MS/S/O
40	1-Nonen-3-ol	21964-44-3	Mushroom	SDE	1558	–	RI/MS/O
41	2-Decanol	1120-06-5	Wax	SAFE	1585	8	RI/MS/S/O
42	1-(Furan-2-yl)propan-1-one	3194-15-8	Grape-like scent, floral, orange blossom	SDE	1597	32	RI/MS/S/O
43	Methyl benzoate	93-58-3	Fruity	SPE	1605	2	RI/MS/S/O
44	Butanoic acid	107-92-6	Rancid, butter-like	SPE	1628	8	RI/MS/S/O
45	2-Furanmethanol	98-00-0	Burnt, caramel, cooked	SDE	1641	4	RI/MS/S/O
46	1-Nonanol	143-08-8	Rose-orange, fatty, bitter	SAFE, SDE	1661	8	RI/MS/S/O
47	Acetophenone	98-86-2	Almonds, flower, meat, must	SDE	1993	2	RI/MS/S/O
48	Salicylaldehyde	90-02-8	Phenolic-almond odor, medicinal	SDE	1699	2	RI/MS/S/O
49	Naphthalene	91-20-3	Plastic, leather	SDE	1706	2	RI/MS/S/O
50	(5-Methyl-2-furyl)methanol	3857-25-8	Caramel-nutty, roasty	SDE	1729	2	RI/MS/S/O
51	Dodecanal	112-54-9	Sweet, floral, somewhat fatty-citrus	SAFE	1710	–	RI/MS/S/O
52	Pentanoic acid	109-52-4	Butter-like odor and burning	SPE	1713	4	RI/MS/S/O
53	Acetamide	60-35-5	Mouse secretion-like	SAFE	1764	4	RI/MS/S/O
54	γ-Heptalactone	105-21-5	Sweet, nut-like, caramel	SDE	1770	–	RI/MS/S/O
55	2,4-Dimethyl-benzaldehyde	15764-16-6	Cherry, almond, spice, vanilla	SDE	1777	–	RI/MS/S/O
56	2,5-Dimethyl-benzaldehyde	5779-94-2	Bitter almond	SPE	1792	32	RI/MS/S/O
57	o-Toluidine	95-53-4	Aniline-like odor	SDE	1794	–	RI/MS/S/O
58	1-Methyl-naphthalene	90-12-0	Earthy, phenolic	SDE	1812	–	RI/MS/S/O
59	Hexanoic acid	142-62-1	Sickening, sweaty, sour, cheesy	SPE	1814	2	RI/MS/S/O
60	α-Methyl-benzenemethanol	98-85-1	Hyacinth–gardenia	SPE	1820	2	RI/MS/S/O
61	1-Furfurylpyrrole	1438-94-4	Cocoa, Green, Roast	SDE	1828	2	RI/MS/S/O
62	Geraniol^a^	106-24-1	Rose	SDE	1827	16	RI/MS/S/O
63	2,3-Dihydropyran-6-one	3393-45-1	Sour	SAFE	1838	–	RI/MS/O
64	Ethyl laurate	106-33-2	Mild fatty-waxy oily, floral fruity	SAFE	1849	2	RI/MS/S/O
65	Geranylacetone	3796-70-1	Fruity	SAFE, SDE	1856	512	RI/MS/S/O
66	Phenylethyl alcohol	60-12-8	Floral, rose-like	SAFE, SDE	1873	8	RI/MS/S/O
67	Benzyl alcohol	100-51-6	Fruity, sweet	SAFE, SDE, SPE	1879	32	RI/MS/S/O
68	Butylated hydroxytoluene	128-37-0	Musty, cresylictype odor	SDE	1889	–	RI/MS/S/O
69	3-Methyl-butanamide	541-46-8	Ammonia	SAFE	1903	2	RI/MS/S/O
70	o-Cresol	95-48-7	Musty, phenolic	SAFE, SDE, SPE	1934	32	RI/MS/S/O
71	Benzothiazole	95-16-9	Nut, Rubber	SAFE	1937	8	RI/MS/S/O
72	Heptanoic acid	111-14-8	Sour sweat-like, fatty odor	SPE	1954	32	RI/MS/S/O
73	Nerolidol	40716-66-3	Floral	SAFE, SDE	2006	32	RI/MS/S/O
74	2-Pentadecanone	2345-28-0	Green	SAFE, SDE	2021	128	RI/MS/S/O
75	Phenol	108-95-2	Phenolic medicinal	SDE, SPE	2033	2	RI/MS/S/O
76	Octanoic acid	124-07-2	Fruity-acid odor	SPE	2050	2	RI/MS/S/O
77	Pyrrole-2-carboxaldehyde	1003-29-8	Musty, beefy, coffee	SDE	2059	16	RI/MS/S/O
78	2-Piperidinone	675-20-7	Ammonia	SAFE	2060	128	RI/MS/S/O
79	Benzalacetone	122-57-6	Creamy, Floral, Pungent	SDE, SPE	2084	16	RI/MS/S/O
80	γ-Decalactone	706-14-9	Coconut-peach like odor, in dilution, peach odor	SDE	2104	4	RI/MS/S/O
81	*cis*-3-Hexenyl benzoate	25152-85-6	Green, herbaceous, woody	SPE	2135	2	RI/MS/S/O
82	2-Phenoxy-Ethanol	122-99-6	Floral	SPE	2139	2	RI/MS/S/O
83	Nonanoic acid	112-05-0	Fatty	SPE	2144	32	RI/MS/S/O
84	Unknown3		Yeasty	SAFE	2160	1024	O
85	α-Bisabolol	515-69-5	Floral, peppery, balsamic	SDE	2175	128	RI/MS/S/O
86	Unknown4		Medicinal, bitter	SAFE	2188	1024	O
87	Methyl palmitate	112-39-0	Oily, waxy, fatty	SAFE, SDE, SPE	2202	8	RI/MS/S/O
88	Palmitoleic acid methyl ester	1120-25-8	Oil	SDE	2225	–	RI/MS/S/O
89	Ethyl palmitate	628-97-7	Waxy sweet odor	SAFE, SDE, SPE	2243	–	RI/MS/S/O
90	Farnesyl acetate	4128-17-0	Oil, Wax	SDE	2253	–	RI/MS/S/O
91	γ-Undecalactone	104-67-6	Apricot, Fruit	SAFE	2259	16	RI/MS/S/O
92	Ethyl 9-hexadecenoate	54546-22-4	Furry	SAFE, SPE	2267	–	RI/MS/O
93	*n*-Decanoic acid	334-48-5	Fatty, unpleasant, rancid	SPE	2279	64	RI/MS/S/O
94	γ-Dodecalactone	2305-05-7	Fatty, fruity, peach odor	SDE	2331	4	RI/MS/S/O
95	Farnesol	4602-84-0	Sweet, oily, linden, muguet floral	SAFE, SDE, SPE	2350	16	RI/MS/S/O
96	Indole	120-72-9	Animalic, cheese notes	SAFE, SDE	2412	64	RI/MS/S/O
97	Benzoic acid	65-85-0	Balsamic	SPE	2412	32	RI/MS/S/O
98	δ-Dodecalactone	713-95-1	Fatty, fruity, peach, buttery	SDE	2427	–	RI/MS/S/O
99	Methyl stearate	112-61-8	Waxy	SPE	2445	4	RI/MS/S/O
100	Benzophenone	119-61-9	Floral	SDE, SPE	2464	2	RI/MS/S/O
101	Octadecanoic acid ethyl ester	111-61-5	Odorless	SPE	2483	–	RI/MS/S/O
102	Tetradecanoic acid	544-63-8	Waxy, oily	SPE	2684	–	RI/MS/S/O
103	Dodecanoic acid	143-07-7	Fatty	SPE	2502	2	RI/MS/S/O
104	Dibutyl phthalate	84-74-2	Aromatic	SPE	2705	–	RI/MS/S/O

RI: retention index (calculated on a DB-WAX column); MS: mass spectrometry (identified by mass spectral library match); S: the reference standard for this compound; O: odor description (aroma character perceived by the panelist).

a Comprehensive two-dimensional gas chromatography (GC × GC) enabled the identification of odorants detected by GC-O that eluded detection by GC–MS.

### 3.3 Aroma-active compounds in YP

**Fig. 4:**
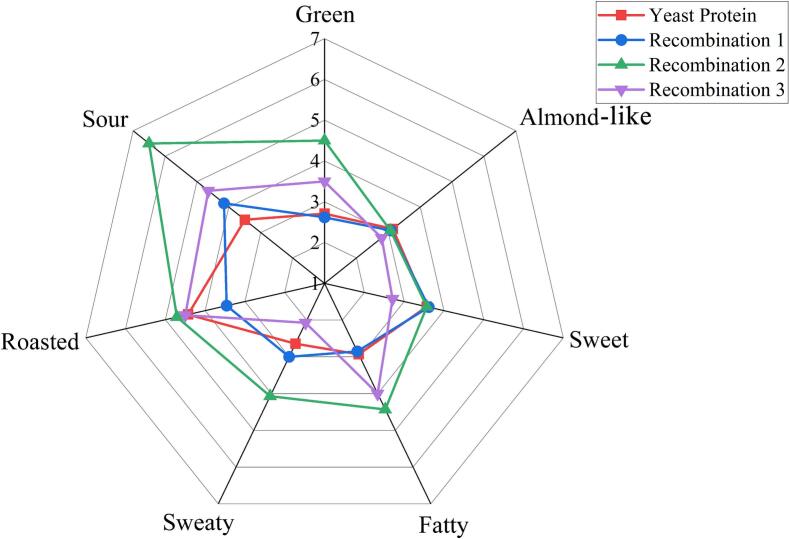
The aroma recombination results of YP (The original YP sample is denoted by the red solid line; recombination 1 (blue solid line) represents the sample reconstituted with volatile compounds extracted through three combined methodologies; recombination 2 (green solid line) corresponds to the sample reconstructed using volatiles isolated via the SAFE coupled with SPE technique; while recombination 3 (purple solid line) indicates the sample regenerated with volatile components obtained through SDE combined with SPE processing.). (For interpretation of the references to color in this figure legend, the reader is referred to the web version of this article.)

As demonstrated in Fig. 4, roasted, sour, sweet, and almond-like attributes are predominant in YP. These sensory profiles showed strong concordance with the GC–MS/O analytical results (Table 1). Notably, the YP extracts contained the highest FD coefficients for compounds characteristic of these aroma descriptors. Seven predominant odorants were characterized as key contributors to the aromatic profile: geranylacetone (FD factor = 512; fruity aroma), 2,6-dimethylpyrazine (FD factor = 256; chocolate, roasted nut, and fried potato), 6-methyl-5-hepten-2-one (FD factor = 128; fatty, green, citrus-like), benzaldehyde (FD factor = 128; bitter almond), 2-pentadecanone (FD factor = 128; green), 2-piperidinone (FD factor = 128; ammonia), and α-bisabolol (FD factor = 128; floral, peppery, balsamic). Among them, five odorants (geranylacetone, 6-methyl-5-hepten-2-one, 2-pentadecanone, 2-piperidinone, and α-bisabolol) were identified for the first time in yeast-derived products. This study represents the first reported identification of these compounds (in addition to 2,6-dimethylpyrazine and benzaldehyde) in YP-derived extracts, expanding our understanding of yeast aromatic components. Geranylacetone, with distinct floral and fruity attributes, could be primarily biosynthesized through three main pathways: carotenoid degradation, oxidation of unsaturated fatty acids, and glycoside hydrolysis (Hao et al., 2023). The presence of 2,6-dimethylpyrazine in YP may originate from microbial metabolic pathways during fermentation. This unstable intermediate undergoes spontaneous oxidative decarboxylation to form diacetyl, which subsequently serves as a metabolic precursor. Through NADH-dependent reductase activity, diacetyl is reduced to acetoin and ultimately to acetaldehyde. These reactive carbonyl compounds then participate in non-enzymatic condensation reactions with amino groups, following the classical Strecker degradation pathway, to generate various pyrazine derivatives including the characteristic 2,6-dimethylpyrazine (Lestari et al., 2023). 6-Methyl-5-hepten-2-one, with distinct fatty, green, citrus-like aromatic profiles, is a biogenic compound synthesized through carbohydrate metabolism via the glycolytic pathway, utilizing 3-phosphoglyceraldehyde and pyruvate as key biosynthetic precursors (Liu et al., 2021). Benzaldehyde, renowned for its distinctive bitter almond-like aroma, arises through the oxidative degradation of aromatic amino acids mediated by the concurrent interplay of carbonyl-amine condensation chemistry and radical-mediated reaction mechanisms (Hidalgo & Zamora, 2019). 2-Pentadecanone, a cyclic ketone compound characterized by its distinctive green aroma, serves as a valuable precursor in flavor and fragrance manufacturing. Recent biochemical studies have revealed that 2-pentadecanone is biosynthesized by yeast strains through the *β*-oxidation pathway of fatty acids (Zhang et al., 2022). 2-Piperidinone, a nitrogen-containing heterocyclic compound characterized by a distinct ammoniacal odor, identified in this study as one of the key factors contributing to the undesirable flavor characteristics in YP products. Despite its significant impact on food quality, the enzymatic pathways governing its biosynthesis remain poorly elucidated, particularly with regard to the specific precursors and regulatory mechanisms involved in microbial metabolic networks. α-Bisabolol, a naturally occurring sesquiterpene alcohol, is primarily identified as a secondary metabolite in the aerial parts of various botanical species (Zhong et al., 2024).

### 3.4 Quantitation analysis

The quantification analysis identified 64 aromatic active compounds with flavor dilution (FD) factors ≥2, which were rigorously quantified using established standard calibration curves. This quantitative framework enabled precise determination of volatile compound concentrations across three orders of magnitude, with correlation coefficients (R^2^) exceeding 0.99 for all calibrated analytes. As detailed in Table 2, specific quantification ions were selected for each compound, accompanied by their corresponding calibration curves. All calibration models demonstrated excellent linearity across the analytical range, with correlation coefficients (R^2^) exceeding 0.99, confirming the reliability of the quantitative measurements. As shown in Table 2, the 2,5-dimethyl-benzaldehyde had the highest concentration (321,387.68 μg/kg) in YP sample, followed by the *n*-decanoic acid (285,751.45 μg/kg), heptanoic acid (150,292.89 μg/kg), α-methyl-benzenemethanol (100,564.47 μg/kg), hexanoic acid (51,855.54 μg/kg), benzoic acid (45,253.67 μg/kg), α-bisabolol (31,276.39 μg/kg), farnesol (21,864.07 μg/kg), dodecanoic acid (16,797.56 μg/kg), benzaldehyde (16,094.71 μg/kg), butanoic acid (15,309.71 μg/kg), ethyl laurate (14,233.09 μg/kg), pentanoic acid (12,493.80 μg/kg), 2-piperidinone (12,229.66 μg/kg), and acetamide (11,411.00 μg/kg). Among the top 15 odorants with the highest concentrations, seven acids associated with undesirable odor characteristics at elevated concentrations, including rancid, cheesy, and sour notes, which may contribute to off-flavors in yeast-derived proteins (Shi et al., 2022). The concentrations of the remaining 49 compounds were all below the 10,000 μg/kg threshold.

**Table 2:** Standard curves and concentrations of aroma-active compounds in YP.

No.	Aroma-active compounds	Qualitative ions	Standard curve	R^2^	concn.(μg/kg)	Threshold value(μg/kg)	OAV
1	2,5-Dimethyl-benzaldehyde	91, 105, 134	*y* = 0.924146*x* + 0.0009	0.9993	321,387.68	200^d^	1607
2	*n*-Decanoic acid	60,129,172	*y* = 0.028588*x* + 0.0004	0.9986	285,751.45	130^d^	2198
3	Heptanoic acid	60,101,130	*y* = 0.127939*x*-0.0017	0.9980	150,292.89	640^d^	235
4	α-Methyl-benzenemethanol	79, 107, 122	*y* = 0.502545*x* + 0.0001	0.9997	100,564.47	479^d^	210
5	Hexanoic acid	60, 87, 116	*y* = 0.199884*x* − 0.0029	0.9980	51,855.54	93^d^	558
6	Benzoic acid	77, 105, 122	*y* = 0.072833*x* − 0.0002	0.9997	45,253.67	47,964.95^e^	<1
7	α-Bisabolol	43, 69, 109	*y* = 0.181642*x* − 0.0001	0.9994	31,276.39	9158.71^e^	3
8	Farnesol	69, 41, 81	*y* = 0.9572*x* − 0.0026	0.9941	21,864.07	1000^d^	22
9	Dodecanoic acid	73, 60, 57	*y* = 0.008367*x* − 0.0001	0.9992	16,797.56	4^d^	4199
10	Benzaldehyde	51, 77, 106	*y* = 0.9696*x* + 0.0004	0.9992	16,094.71	320^d^	50
11	Butanoic acid	60, 73, 88	*y* = 0.185866*x* − 0.0027	0.9979	15,309.71	2400^a^	6
12	Ethyl laurate	70, 88, 101	*y* = 0.1099*x* − 0.0001	0.9976	14,233.09	5900^e^	2
13	Pentanoic acid	60, 73, 100	*y* = 0.295318*x* − 0.0044	0.9980	12,493.80	11,000^a^	1
14	2-Piperidinone	42, 55, 99	*y* = 0.5296*x* − 0.0001	0.9983	12,229.66	2.43^d^	5033
15	Acetamide	59, 44, 28	*y* = 0.3416*x* − 0.0001	0.9904	11,411.00	140,000^d^	<1
16	o-Cresol	108, 79, 90	*y* = 0.7962*x* + 0.0001	0.9997	9207.53	3.9^d^	2361
17	Benzyl alcohol	77, 79, 108	*y* = 0.9628*x* + 0.0001	0.9998	8528.87	2546.21^d^	3
18	Geranylacetone	43, 69, 151	*y* = 0.581184*x* − 0.0014	0.9991	8488.52	60^d^	141
19	Nonanal	29, 41, 57	*y* = 0.6077*x* + 0.0003	0.9967	6990.87	2.8^a^	2497
20	Phenylethyl Alcohol	91, 122, 65	*y* = 1.081661*x* − 0.0008	0.9993	4536.07	140^d^	32
21	1-Nonanol	56, 70, 43	*y* = 0.8861*x* − 0.0003	0.9998	4110.24	45.5^d^	90
22	2-Furanmethanol	98, 81, 53	*y* = 3.788813*x* − 0.0030	0.9998	3469.17	1900^d^	2
23	Methyl benzoate	77, 105, 136	*y* = 8.0907*x* + 0.0089	0.9962	3291.66	0.52^e^	6330
24	2-Decanol	41, 45, 55	*y* = 0.9998*x* − 0.0005	0.9995	2994.74	25^d^	120
25	Hexanal	44, 56, 27	*y* = 0.0957*x* + 0.0106	0.9998	2925.32	2.4^a^	1219
26	Octanoic acid	55, 73, 101	*y* = 5.022481*x* − 0.0127	0.9995	2877.88	910^d^	3
27	2,6-Dimethyl-pyrazine	108, 67, 42	*y* = 0.926193*x* + 0.0013	0.9997	2190.95	718^d^	3
28	γ-Decalactone	85, 128, 29	*y* = 1.634162*x* + 0.0062	0.9980	2181.02	1.1^a^	1983
29	2-Pentadecanone	58, 43, 85	*y* = 0.690914*x* − 0.0026	0.9996	2121.87	1^d^	2122
30	3-Methyl-butanamide	44, 59, 86	*y* = 0.8658*x* − 0.0001	0.9999	2006.52	1374.75^e^	1
31	Phenol	39, 66, 94	*y* = 0.992290*x* + 0.0048	0.9990	1986.89	31^d^	64
32	*cis*-3-Hexenyl benzoate	67, 105, 123	*y* = 1.031419*x* + 0.0004	0.9993	1911.01	656.45^e^	3
33	6-Methyl-5-hepten-2-one	43, 55, 69	*y* = 0.3709*x* + 0.0003	0.9997	1500.43	68^d^	22
34	Nerolidol	69, 41, 93	*y* = 4.377485*x* − 0.0428	0.9994	1444.32	250^d^	6
35	1-Octen-3-ol	43, 57, 72	*y* = 1.086*x* + 0.0001	1.0000	1435.67	14^b^	103
36	γ-Undecalactone	29, 55, 85	*y* = 1.5255*x* − 0.0015	0.9950	1402.38	2.1^a^	668
37	(5-Methyl-2-furyl)methanol	95, 112, 53	*y* = 0.235552*x* − 0.0001	0.9997	1323.75	4485.32^e^	<1
38	Indole	63, 90, 117	*y* = 2.2669*x* + 0.0001	0.9999	1310.63	11^d^	119
39	Acetophenone	105, 77, 120	*y* = 1.160218*x* + 0.0002	0.9994	1125.01	65^d^	17
40	2-Nonanone	58, 43, 142	*y* = 1.4805*x* + 0.0002	0.9995	1026.53	200^b^	5
41	2-Octanone	43, 58, 71	*y* = 1.5995*x* + 0.0051	0.9989	986.95	50.2^d^	20
42	Methyl palmitate	74, 87, 55	*y* = 0.807214*x* + 0.0012	0.9994	946.78	2000^d^	<1
43	Octanal	41, 57, 84	*y* = 0.5672*x* + 0.0001	0.9930	942.29	3.4^a^	277
44	Salicylaldehyde	122, 65, 39	*y* = 0.732155*x* − 0.0038	0.9991	870.65	30^d^	29
45	Propanoic acid	28, 45, 74	*y* = 0.886819*x* + 0.0005	0.9995	776.35	100^d^	8
46	2-Phenoxy-ethanol	77, 94, 138	*y* = 1.237755*x* + 0.0004	0.9993	671.88	600,687.31^e^	<1
47	Ethylbenzene	91, 106, 77	*y* = 1.966965*x* + 0.0008	0.9995	584.76	29^c^	244
48	Benzothiazole	135, 108, 69	*y* = 0.78624*x* + 0.0001	1.0000	431.08	80^d^	5
49	Disulfide dimethyl	94, 45, 79	*y* = 0.2679*x* − 0.0224	0.9999	381.21	1.2^b^	318
50	2-Decanone	58, 43, 71	*y* = 8.327059*x* + 0.0038	0.9999	300.50	3^d^	100
51	γ-Dodecalactone	85, 29, 41	*y* = 1.142254*x* − 0.0003	0.9997	264.29	0.43^a^	615
52	1-Furfurylpyrrole	81, 53, 147	*y* = 0.0412*x* + 0.0023	0.9976	170.71	100^d^	2
53	Naphthalene	128, 102, 64	*y* = 4.167260*x* + 0.0016	0.9996	162.37	1^d^	162
54	Methyl stearate	74, 87, 298	*y* = 7.391232*x* + 0.0127	0.9928	106.70	236,125.32^e^	<1
55	Nonanoic acid	60, 73, 57	*y* = 2.318771*x* − 0.0467	0.9980	98.75	3000^d^	<1
56	3-Ethyl-2,5-dimethyl-pyrazine	135, 108, 42	*y* = 6.148378*x* + 0.0181	0.9995	80.91	8.6^d^	9
57	4-Cyclopentene-1,3-dione	96, 42, 54	*y* = 1.528371*x* + 0.0020	0.9993	70.84	150,309.88^e^	<1
58	4-Methyl-pyrimidine	94, 67, 53	*y* = 5.064300*x* − 0.3042	0.9996	51.96	4651.23^e^	<1
59	1-Butanol	56, 31, 41	*y* = 0.5134*x* − 0.0033	0.9999	51.61	590^a^	<1
60	2-Propionylfuran	95, 124, 39	*y* = 0.2117*x* − 0.0068	0.9993	30.95	6764.40^e^	<1
61	Pyrrole-2-carboxaldehyde	95, 66, 39	*y* = 3.919571*x* + 0.0006	0.9998	23.29	65.000^e^	<1
62	Benzalacetone	131, 103, 146	*y* = 0.946822*x* − 0.0002	0.9998	21.34	2778.04^e^	<1
63	Geraniol	69, 41, 123	*y* = 1.075220*x* + 0.0043	0.9995	14.7	6.6^d^	2
64	Benzophenone	105, 77, 182	*y* = 0.3399*x* − 0.0067	0.9996	11.00	8007.07^e^	<1

a The reference for odor threshold comes from (Czerny et al., 2008)

b The reference for odor threshold comes from (Zhang et al., 2018)

c The reference for odor threshold comes from (Zhu et al., 2018)

d The reference for odor threshold comes from a book named Odor thresholds compilations of odor threshold values in air, water and other media (second enlarged and revised edition)

e The odor threshold data was determined in this study, as shown in Fig. 3.

**Fig. 3:**
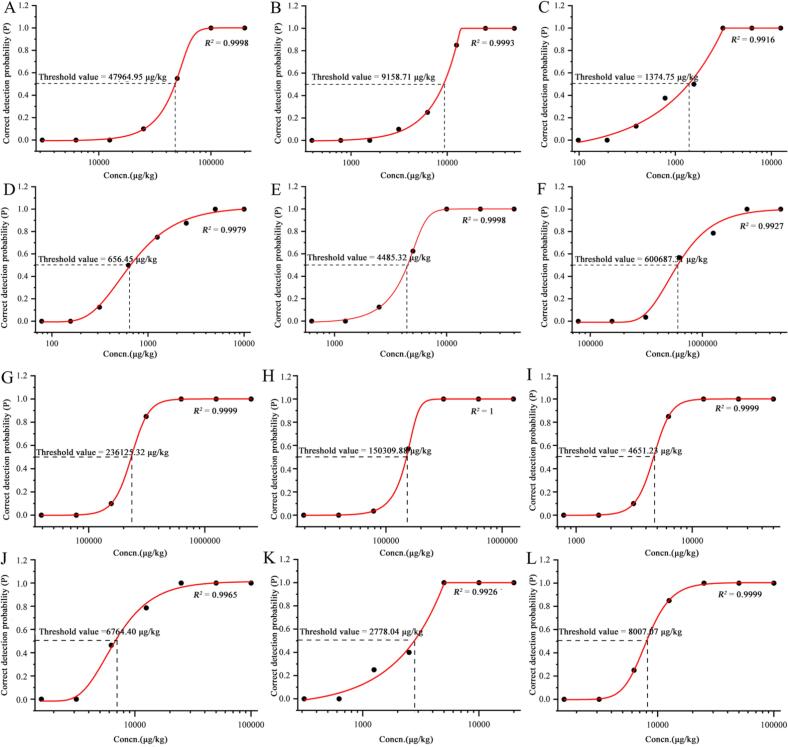
Determination of Odor Threshold S-Curves (A: Benzoic acid; B: α-Bisabolol; C: 3-Methyl-butanamide; *D*: cis-3-Hexenyl benzoate; E: (5-Methyl-2-furyl)methanol; F: 2-Phenoxy-ethanol; G: Methyl stearate; H: 4-Cyclopentene-1,3-dione; I: 4-Methyl-pyrimidine; J: 2-Propionylfuran; K: Benzalacetone; L: Benzophenone).

### 3.5 Calculation of odor activity value

Based on the quantitative results (Table 2), the OAV of each aroma-active compounds were detected. The results revealed that 50 compounds exhibiting OAV > 1, with 33 of them having OAV values >10, indicating that these compounds were key contributors to YP aroma profiles. This observation clearly demonstrates that compound concentration alone does not necessarily correlate with aroma impact, as exemplified by three notable cases: benzoic acid, 3-methyl-butanamid and acetamide. Despite concentrations exceeding 10,000 μg/kg, benzoic acid, 3-methyl-butanamide, and acetamide showed negligible flavor contributions, with their OAV lower than 1. This apparent paradox between chemical abundance and sensory impact underscores the importance of odor threshold considerations in aroma compound evaluation. The chromatographic data summarized in Table 3 substantiated the initial GC-O characterization, wherein a majority of aroma-active compounds (≥85% OAV > 1) displayed proportional enhancement in sensory intensity corresponding to their odor activity values. Several volatile compounds in YP sample exhibited remarkably high OAV, as detailed in Table 2. Methyl benzoate demonstrated the most prominent OAV of 6330, characterized by fruity attributes, followed by 2-piperidinone (OAV = 5033) imparted ammonia-related aromas, dodecanoic acid (4199) associated with fatty-waxy characteristics, nonanal (2497) contributing distinct fatty and citrus, o-cresol (2361) contributing musty phenolic notes, and *n*-decanoic acid (2198) known for its fatty, unpleasant, rancid. The overall flavor profile was further defined by several key odorants, each contributing distinct aromatic nuances. γ-Decalactone (1983) imparted a coconut-peach character that became more peach-like upon dilution. Hexanal (1219) contributed complex fatty, green, and fruity tones. The profile was also shaped by the green vegetal notes of 2-pentadecanone (2122) and the bitter almond nuances of 2,5-dimethyl-benzaldehyde (1607). Dimethyl disulfide (318) added sulfurous, cabbage-like vegetable notes. Notably, octanal (277) exhibited concentration-dependent olfaction: fatty-citrus at standard levels and honeyed when diluted. Finally, 1-octen-3-ol (103) completed the spectrum with prominent fungal or mushroom undertones.

**Table 3:** Omission tests results of YP.

No.	Aroma qualities	Omitted compounds	Correct number	Significance	Aroma difference
1	Bitter almond	2,5-Dimethyl-benzaldehyde	7/12	NS	–
2	Fatty, rancid	*n*-Decanoic acid	10/12	*	Sour↓, sweet↑
3	Sour sweat-like, fatty	Heptanoic acid	6/12	NS	–
4	Hyacinth–gardenia	α-Methyl-benzenemethanol	8/12	NS	–
5	Sweaty, cheesy	Hexanoic acid	10/12	*	Green↑, sweaty↓
6	Fatty, waxy	Dodecanoic acid	7/12	NS	–
7	Bitter almond	Benzaldehyde	10/12	*	Almond-like↓
8	Fruit	Methyl benzoate	10/12	*	Sweaty↑
9	Floral, rose-like	Phenylethyl Alcohol	7/12	NS	–
10	Ammonia	2-Piperidinone	8/12	NS	–
11	Fruit	Geranylacetone	10/12	*	Sweaty↑
12	Musty, phenolic	o-Cresol	5/12	NS	–
13	Fatty, citrus-like	Nonanal	4/12	NS	–
14	Rose-orange, fatty, bitter	1-Nonanol	10/12	*	Sweet↑
15	Wax	2-Decanol	10/12	*	Sour↑
16	Peach odor	γ-Decalactone	7/12	NS	–
17	Green	2-Pentadecanone	10/12	*	Green↓
18	Phenolic medicinal	Phenol	8/12	NS	–
19	Sweet, muguet floral	Farnesol	10/12	*	Sweet↓, sweaty↑
20	Floral animalic, cheese notes	Indole	7/12	NS	–
21	Fatty, green, citrus-like	6-Methyl-5-hepten-2-one	6/12	NS	–
22	Mushroom	1-Octen-3-ol	7/12	NS	–
23	Apricot, Fruit	γ-Undecalactone	10/12	*	Sweet↑
24	Almonds, flower, meat, must	Acetophenone	7/12	NS	–
25	Floral, green, fruity	2-Octanone	7/12	NS	–
26	Fatty, citrus	Octanal	10/12	*	Fatty↓, green↓
27	Fatty, green, grassy, fruity	Hexanal	3/12	NS	–
28	Fragrant	Ethylbenzene	3/12	NS	–
29	Cabbage, garlic, onion	Disulfide, dimethyl	6/12	NS	–
30	Floral	2-Decanone	8/12	NS	–
31	Fatty, fruity, peach odor	γ-Dodecalactone	6/12	NS	–
32	Coal tar odor	Naphthalene	7/12	NS	–
33	Phenolic-almond odor, medicinal	Salicylaldehyde	5/12	NS	–

NS, no signifcant difference; *, significance difference at *p* < 0.05, −, no aroma difference.

### 3.6 Aroma recombination and omission

The aroma recombination process involved dissolving odor-active compounds (OAV > 10) into an odorless ultrapure solution. These compounds were subsequently incorporated into a deodorized matrix at concentrations equivalent to those detected in the original samples to construct YP aroma recombinants. Recombination models 1, 2, and 3 correspond to the volatile compounds obtained from the combination of SAFE, SDE and SPE, the combination of SAFE and SPE, and the combination of SDE and SPE, respectively. As shown in Fig. 4, comparative analysis of aroma profiles indicated that recombination 1 had the highest sensory similarity of 90% to the real YP sample. Notably, while the recombination model exhibited strong congruence with original samples, subtle sensory variations were observed. The roasted note was marginally attenuated compared to native YP, whereas sour and sweaty characteristics showed slight intensification in the recombinant formulation. These minor discrepancies may be attributed to either undetected synergistic/antagonistic interactions between aroma components or potential matrix effects influencing compound volatility.

To validate the contribution of specific compounds, those with OAV >10 were screened, ultimately establishing 33 omission analytical models. The results (Table 3) revealed a significant difference (*p* < 0.05) in almond-like attributes intensity upon omission of benzaldehyde, confirming its role as the primary odorant responsible for almond-like attributes in YP. Additionally, the omission of *n*-decanoic acid (*p* < 0.05) or 1-nonanol (*p* < 0.05) significantly enhanced sweet attributes perception, suggesting that both compounds act as inhibitors of sweet attributes. Sweaty attributes intensity was markedly reduced in the absence of hexanoic acid (*p* < 0.05), identifying hexanoic acid as the dominant contributors to sweaty attributes in YP. Conversely, omitting geranylacetone (*p* < 0.05), farnesol (*p* < 0.05), or methyl benzoate (*p* < 0.05) resulted in significantly intensified sweaty attributes and diminished sweet attributes, indicating that these three compounds possess inherent sweet aromatic properties and suppress sweat attributes. The omission of γ-undecalactone (*p* < 0.05) led to a pronounced enhancement of sweet attributes, implying its synergistic interaction with compounds generating unpleasant odors. Moreover green attributes intensity was significantly reduced upon removal of 2-pentadecanone (*p* < 0.05) or octanal (*p* < 0.05), establishing these compounds as key contributors to green aromatic notes in YP. Finally, the removal of 2-decanol (*p* < 0.05) was associated with an increase in sourness intensity. One plausible explanation is a synergistic effect: while 2-decanol contributes minimal wax odor alone, its presence may interact with acids to suppress sourness perception. In contrast, no significant aroma differences were detected when the remaining 22 compounds were present (*p* > 0.05), indicating that these compounds constitute non-essential odor components for the overall aroma profile of YP. In summary, the following compounds were identified as key aroma components in YP: benzaldehyde, *n*-decanoic acid, hexanoic acid, methyl benzoate, geranylacetone, 1-nonanol, 2-decanol, 2-pentadecanone, farnesol, γ-undecalactone, and octanal.

## 4 Conclusion

One hundred odor-active compounds were identified in the YP samples through the combined use of three extraction techniques, analyzed by GC–MS/O and evaluated via AEDA. These compounds were sensorially categorized into four primary descriptors: green, fatty, sweet, and roasted attributes. Quantitative analysis identified several compounds with significant OAVs (>100), including methyl benzoate, which demonstrated the highest potency (OAV = 6330). Subsequent compounds of significance included 2-piperidinone (5033), dodecanoic acid (4199), nonanal (2497), o-cresol (2361), *n*-decanoic acid (2198), 2-pentadecanone (2122), γ-decalactone (1983), 2,5-dimethyl-benzaldehyde (1607), dimethyl disulfide (318), octanal (277), and 1-octen-3-ol (103), establishing their roles as key contributors to the overall aroma profile. The results indicated that SAFE and SDE were more effective for bulk recovery, accounting for over 80% of the extracted YP mass. Conversely, SPE offered superior performance in the targeted isolation of characteristic organic acids. Notably, GC × GC-TOF-MS analysis effectively complemented conventional GC–MS by identifying additional compounds beyond standard detection limits. Recombination experiments confirmed that integrated extracts from the three methods achieved optimal sensory fidelity to native YP (90%). Omission experiments further confirmed that benzaldehyde, *n*-decanoic acid, hexanoic acid, methyl benzoate, geranylacetone, 1-nonanol, 2-decanol, 2-pentadecanone, farnesol, γ-undecalactone, and octanal were key aroma components in YP. This study elucidated the principal flavor components in YP and established an analytical framework for characterizing YP flavor, providing valuable insights for the development and quality control of yeast-based protein products. This study systematically characterized the volatile flavor profiles of yeast proteins using three extraction methods: SDE, SPE, and SAFE. SDE excels at extracting trace volatile compounds from samples; SPE is better suited for the separation and extraction of organic acids; and SAFE is ideal for characterizing thermally unstable and easily oxidizable aromatic compounds. The results revealed method dependent differences in the detection of flavor providing a practical basis for selecting appropriate extraction techniques according to specific research or production goals. From a practical perspective, these findings directly inform the food application of yeast proteins by enabling targeted flavor regulation, thereby reinforcing the industrial significance of method selection. Collectively, these conclusions offer actionable guidance for flavor-driven innovation in yeast protein-based foods and for method selection in future flavor analysis studies.

## Acknowledgements and fundings

This work was supported by Beijing Life Science Academy (BLSA) [No. 2023600CA0080], R&D Program of Beijing Municipal Education Commission [No. KM202410011008], and 10.13039/501100001809National Natural Science Foundation of China [No. 32102118].

## CRediT authorship contribution statement

**Jiahui Chen:** Writing – review & editing, Writing – original draft, Methodology, Investigation. **Boya Cao:** Investigation, Data curation. **Zikang Xu:** Investigation, Data curation. **Baoguo Sun:** Writing – review & editing, Resources. **Shihao Sun:** Writing – review & editing. **Dandan Pu:** Writing – review & editing, Methodology, Investigation. **Lili Zhang:** Writing – review & editing, Investigation. **Yuyu Zhang:** Writing – review & editing.

## Declaration of competing interest

The authors declare that they have no known competing financial interests or personal relationships that could have appeared to influence the work reported in this paper.

## Data Availability

Data will be made available on request.

## Appendix A Supplementary data

Supplementary material 1 shows the volatile compounds detected by comprehensive GC × GC-TOF-MS.
