# Supplementary material for: Decoding and reconstructing yeast protein flavor based on an integrated sensory-omics approach
Source: Food Chem X. 2026 Jun 9;37:104060. doi: 10.1016/j.fochx.2026.104060 (PMC13293764; doi:10.1016/j.fochx.2026.104060)
Supplement: Supplementary file 1 — Supplementary material 1 shows the volatile compounds detected by comprehensive GC × GC-TOF-MS. [file mmc1.docx]

Table S1. Identification of the aroma-active compounds by GC×GC-TOF-MS in yeast proteins.

| No. | Volatile compounds | CAS | Chemical formula | RI | NIST-RI | DB-WAX-RI | Concentration (μg/kg) |
| --- | --- | --- | --- | --- | --- | --- | --- |
| 1 | 3-Methyl-butanal | 590-86-3 | C5H10O | 0 | 652-S | 916 | 59.85±13.58 |
| 2 | Pentanal | 110-62-3 | C5H10O | 700 | 699-S | 984 | 89.69±19.39 |
| 3 | Toluene | 108-88-3 | C7H8 | 764 | 763-S | 1036 | 41.47±4.84 |
| 4 | Ethyl isovalerate | 108-64-5 | C7H14O2 | 849 | 854-S | 1064 | 52.22±6.40 |
| 5 | Dimethyl disulfide | 624-92-0 | C2H6S2 | 742 | 746-S | 1071 | 56.98±7.95 |
| 6 | 2-Hexanone | 591-78-6 | C6H12O | 786 | 790-S | 1075 | 19.62±4.05 |
| 7 | Hexanal | 66-25-1 | C6H12O | 800 | 800-S | 1097 | 247.17±23.32 |
| 8 | p-Xylene | 106-42-3 | C8H10 | 894 | 865-S | 1119 | 663.37±165.84 |
| 9 | Ethylbenzene | 100-41-4 | C8H10 | 859 | 855-S | 1123 | 239.84±23.46 |
| 10 | 1,3,5-Trioxane | 110-88-3 | C3H6O3 |  | 658-S | 1167 | 479.95±131.48 |
| 11 | o-Xylene | 95-47-6 | C8H10 | 868 | 887-S | 1175 | 610.63±243.39 |
| 12 | 2-Heptanone | 110-43-0 | C7H14O | 887 | 891-S | 1180 | 113.35±13.59 |
| 13 | Heptanal | 111-71-7 | C7H14O | 901 | 901-S | 1182 | 364.16±24.11 |
| 14 | 4-Octanone | 589-63-9 | C8H16O | 933 | 976-S | 1224 | 517.99±165.35 |
| 15 | 2-pentyl-furan | 3777-69-3 | C9H14O | 989 | 993-S | 1229 | 67.49±5.50 |
| 16 | 1-Pentanol | 71-41-0 | C5H12O | 759 | 765-S | 1241 | 22.42±3.19 |
| 17 | Styrene | 100-42-5 | C8H8 | 893 | 893-S | 1254 | 344.59±40.09 |
| 18 | 1,2,4-trimethyl-benzene | 95-63-6 | C9H12 | 995 | 990-S | 1267 | 78.25±12.41 |
| 19 | Acetoin | 513-86-0 | C4H8O2 | 708 | 713-S | 1273 | 14.08±4.21 |
| 20 | Cyclohexanone | 108-94-1 | C6H10O | 896 | 894-S | 1281 | 161.37±49.57 |
| 21 | Octanal | 124-13-0 | C8H16O | 1003 | 1003-S | 1286 | 157.53±8.35 |
| 22 | 2-Octanone | 111-13-7 | C8H16O | 988 | 990-S | 1297 | 135.64±14.42 |
| 23 | 1-Ethyl-3,5-dimethyl-benzene | 934-74-7 | C10H14 | 1075 | 1058-S | 1321 | 69.15±32.30 |
| 24 | 2,5-Dimethyl-pyrazine | 123-32-0 | C6H8N2 | 912 | 917-S | 1321 | 179.36±33.16 |
| 25 | 6-Methyl-5-hepten-2-one | 110-93-0 | C8H14O | 983 | 986-S | 1323 | 62.33±6.40 |
| 26 | 4-Methyl-pyrimidine | 3438-46-8 | C5H6N2 | 824 | 853-S | 1328 | 51.96±6.35 |
| 27 | 1-Hexanol | 111-27-3 | C6H14O | 863 | 868-S | 1345 | 45.52±4.56 |
| 28 | 2-Ethyl-6-methyl-pyrazine | 13925-03-6 | C7H10N2 | 997 | 1003-S | 1375 | 192.13±16.31 |
| 29 | Dimethyl trisulfide | 3658-80-8 | C2H6S3 | 970 | 970-S | 1378 | 227.31±47.30 |
| 30 | 2-Nonanone | 821-55-6 | C9H18O | 1090 | 1092-S | 1387 | 85.55±5.44 |
| 31 | Nonanal | 124-19-6 | C9H18O | 1104 | 1104-S | 1390 | 283.68±20.62 |
| 32 | Linalool oxide | 5989-33-3 | C10H18O | 1070 | 1074-S | 1425 | 76.51±52.28 |
| 33 | 3-Ethyl-2,5-dimethyl-pyrazine | 13360-65-1 | C8H12N2 | 1074 | 1082-S | 1430 | 80.91±6.60 |
| 34 | 2-Octenal | 2548-87-0 | C8H14O | 1057 | 1060-S | 1437 | 9.52±2.31 |
| 35 | Ethyl octanoate | 106-32-1 | C10H20O | 1195 | 1196-S | 1440 | 6.24±1.15 |
| 36 | 1-Heptanol | 111-70-6 | C7H16O | 966 | 970-S | 1447 | 115.91±20.21 |
| 37 | 1-Octen-3-ol | 3391-86-4 | C8H16O | 977 | 980-S | 1458 | 8.01±0.39 |
| 38 | 1,2,4,5-Tetramethyl-benzene | 95-93-2 | C10H14 | 1116 | 1116-S | 1459 | 155.74±46.17 |
| 39 | 2-Cyclohexen-1-one | 930-68-7 | C6H8O | 931 | 920-S | 1462 | 33.20±0.00 |
| 40 | 2-Ethyl-3,5-dimethyl-pyrazine | 13925-07-0 | C8H12N2 | 1074 | 1084-S | 1464 | 84.65±0.00 |
| 41 | Decanal | 112-31-2 | C10H20O | 1206 | 1206-S | 1472 | 27.77±5.35 |
| 42 | Benzene, 1,2-dichloro- | 95-50-1 | C6H4Cl2 | 1033 | 1043-S |  | 752.01±51.67 |
| 43 | 2-Decanone | 693-54-9 | C10H20O | 1191 | 1193-S | 1482 | 90.11±9.57 |
| 44 | 2-Ethyl-1-hexanol | 104-76-7 | C8H18O | 1026 | 1030-S | 1484 | 104.70±5.95 |
| 45 | Benzaldehyde | 100-52-7 | C7H6O | 961 | 962-S | 1502 | 5961.21±491.77 |
| 46 | 1,2,3,4-Tetrahydro-naphthalen | 119-64-2 | C10H12 | 1162 | 1155-S | 1525 | 31.79±12.95 |
| 47 | 2-Nonanol | 628-99-9 | C9H20O | 1101 | 1102-S | 1530 | 8.06±1.01 |
| 48 | Linalool | 78-70-6 | C10H18O | 1099 | 1099-S | 1547 | 14.93±1.96 |
| 49 | 1-Nonen-3-ol | 21964-44-3 | C9H18O | 1079 | 1080-S | 1555 | 4.43±0.50 |
| 50 | 1-Octanol | 111-87-5 | C8H18O | 1068 | 1071-S | 1558 | 60.32±6.77 |
| 51 | 6-Methyl-3,5-heptadiene-2-one | 1604-28-0 | C8H12O | 1102 | 1107-S | 1582 | 7.59±0.94 |
| 52 | 2-Nonenal | 18829-56-6 | C9H16O | 1160 | 1162-S | 1582 | 14.70±2.12 |
| 53 | 2-Undecanone | 112-12-9 | C11H22O | 1292 | 1294-S | 1599 | 14.90±1.43 |
| 54 | 2-Methyl-benzaldehyde | 529-20-4 | C8H8O | 1067 | 1064-S | 1621 | 46.55±2.37 |
| 55 | Undecanal | 112-44-7 | C11H22O | 1308 | 1307-S | 1622 | 9.34±2.03 |
| 56 | Acetophenone | 98-86-2 | C8H8O | 1065 | 1065-S | 1627 | 19.71±0.00 |
| 57 | 2-Decenal | 3913-81-3 | C10H18O | 1263 | 1263-S | 1630 | 7.49±0.44 |
| 58 | 2-Furanmethanol | 98-00-0 | C5H6O2 | 847 | 859-S | 1635 | 253.01±162.26 |
| 59 | Ethyl decanoate | 110-38-3 | C12H24O2 | 1393 | 1396-S | 1638 | 17.55±1.29 |
| 60 | Levomenthol | 2216-51-5 | C10H20O | 1178 | 1175-S | 1639 | 347.72±0.08 |
| 61 | 1-Nonanol | 143-08-8 | C9H20O | 1170 | 1173-S | 1640 | 119.98±15.82 |
| 62 | 2,5-dimethyl-benzaldehyde | 5779-94-2 | C9H10O | 1216 | 1208-S | 1705 | 1274.67±283.89 |
| 63 | Naphthalene | 91-20-3 | C10H8 | 1186 | 1182-S | 1707 | 273.69±61.00 |
| 64 | Dodecanal | 112-54-9 | C12H24O | 1409 | 1409-S | 1710 | 32.69±13.62 |
| 65 | Citronellol | 106-22-9 | C10H20O | 1226 | 1228-S | 1750 | 16.87±2.49 |
| 66 | gamma-Heptalactone | 105-21-5 | C7H12O2 | 1149 | 1159-S | 1764 | 27.78±0.00 |
| 67 | Nerol | 106-25-2 | C10H18O | 1224 | 1228-S | 1767 | 10.96±1.61 |
| 68 | o-Toluidine | 95-53-4 | C7H9N | 1069 | 1070-S | 1789 | 874.10±18.53 |
| 69 | Tridecanal | 10486-19-8 | C13H26O | 1511 | 1512-S | 1800 | 17.58±3.09 |
| 70 | Nerylacetone | 3879-26-3 | C13H22O | 1428 | 1435-S | 1813 | 9.24±0.00 |
| 71 | 2-Tridecanone | 593-08-8 | C13H26O | 1495 | 1497-S | 1814 | 18.34±3.20 |
| 72 | 3-Methyl-benzenamine | 108-44-1 | C7H9N | 1069 | 1075-S | 1831 | 722.19±0.00 |
| 73 | Geraniol | 106-24-1 | C10H18O | 1249 | 1255-S | 1841 | 14.70±1.77 |
| 74 | Ethyl laurate | 106-33-2 | C14H28O | 1592 | 1595-S | 1850 | 43.39±2.79 |
| 75 | 6,10-Dimethyl-5,9-undecadien-2-one | 3796-70-1 | C13H22O | 1428 | 1453-S | 1856 | 9.95±0.55 |
| 76 | 1-Methyl-naphthalene | 90-12-0 | C11H10 | 1296 | 1307-S | 1875 | 146.81±74.55 |
| 77 | Benzyl alcohol | 100-51-6 | C7H8O | 1032 | 1036-S | 1877 | 2296.47±537.03 |
| 78 | 3-Ethyl-2-hydroxy-2-cyclopenten-1-one | 21835-01-8 | C7H10O2 | 1089 | 1106-S | 1894 | 78.14±8.54 |
| 79 | gamma-Octanoic lactone | 104-50-7 | C8H14O2 | 1254 | 1261-S | 1898 | 35.17±5.40 |
| 80 | Phenylethyl Alcohol | 60-12-8 | C8H10O | 1112 | 1116-S | 1912 | 646.88±127.53 |
| 81 | Tetradecanal | 124-25-4 | C14H28O | 1612 | 1613-S | 1930 | 27.98±6.22 |
| 82 | Benzothiazole | 95-16-9 | C7H5NS | 1226 | 1229-S | 1942 | 135.06±20.68 |
| 83 | 1-Dodecanol | 112-53-8 | C12H26O | 1474 | 1473-S | 1964 | 22.95±8.22 |
| 84 | o-Cresol | 95-48-7 | C7H8O | 1048 | 1054-S | 1979 | 2171.77±257.41 |
| 85 | Phenol | 108-95-2 | C6H6O | 973 | 980-S | 2007 | 143.11±54.75 |
| 86 | Nerolidol | 142-50-7 | C15H26O | 1530 | 1544-S | 2005 | 13.06±0.47 |
| 87 | gamma-Nonanolactone | 104-61-0 | C9H16O2 | 1358 | 1363-S | 2011 | 70.02±23.64 |
| 88 | trans-Nerolidol | 40716-66-3 | C15H26O | 1560 | 1564-S | 2017 | 336.12±40.17 |
| 89 | 2-Pentadecanone | 2345-28-0 | C15H30O | 1697 | 1698-S | 2021 | 65.60±7.42 |
| 90 | Pentadecanal | 2765-11-9 | C15H30O | 1714 | 1715-S | 2054 | 31.83±8.60 |
| 91 | Ethyl myristate | 124-06-1 | C16H32O | 1791 | 1794-S | 2054 | 28.60±3.72 |
| 92 | Cedrol | 77-53-2 | C15H26O | 1612 | 1598-S | 2106 | 114.81±3.61 |
| 93 | gamma-Decalactone | 706-14-9 | C10H18O2 | 1464 | 1470-S | 2126 | 402.82±58.22 |
| 94 | α-Bisabolol | 515-69-5 | C15H26O | 1686 | 1684-S | 2175 | 244.54±0.00 |
| 95 | Palmitic acid ethyl ester | 628-97-7 | C18H36O | 1992 | 1993-S | 2220 | 151.96±42.47 |
| 96 | 2-Aminoacetophenone | 551-93-9 | C8H9NO | 1301 | 1308-S | 2223 | 59.92±0.00 |
| 97 | 3,4'-Diethyl-1,1'-biphenyl | 61141-66-0 | C16H18 | 1674 | 1692-S | 2228 | 154.58±30.01 |
| 98 | Undecan-4-olide | 104-67-6 | C11H20O | 1570 | 1576-S | 2238 | 69.62±0.83 |
| 99 | 2-Heptadecanone | 2922-51-2 | C17H34O | 1900 | 1904-S | 2255 | 55.75±11.63 |
| 100 | Ethyl 9-hexadecenoate | 54546-22-4 | C18H34O | 1970 | 1977-S | 2283 | 383.50±36.27 |
| 101 | Dimethyl phthalate | 131-11-3 | C10H10O | 1446 | 1454-S | 2325 | 199.75±87.05 |
| 102 | 2,4-Di-tert-butylphenol | 96-76-4 | C14H22O | 1505 | 1519-S | 2330 | 2289.97±628.45 |
| 103 | Farnesyl acetate | 4128-17-0 | C17H28O2 | 1830 | 1843-S | 2334 | 57.29±16.37 |
| 104 | CIS-trans-Farnesol | 3790-71-4 | C15H26O | 1691 | 1697-S | 2355 | 81.17±18.11 |
| 105 | γ-Dodecalactone | 2305-05-7 | C12H22O | 1676 | 1678-S | 2366 | 353.43±55.91 |
| 106 | Farnesol | 4602-84-0 | C15H26O | 1714 | 1713-S | 2378 | 454.37±40.71 |
| 107 | δ-Dodecalactone | 713-95-1 | C12H22O | 1703 | 1719-S | 2420 | 60.75±0.00 |
| 108 | Indole | 120-72-9 | C8H7N | 1291 | 1295-S | 2448 | 946.48±239.40 |
| 109 | Benzophenone | 119-61-9 | C13H10O | 1628 | 1635-S | 2457 | 87.26±0.00 |
| 110 | Ethyl Oleate | 111-62-6 | C20H38O2 | 2166 | 2173-S | 2470 | 183.92±41.87 |
| 111 | Ethyl stearate | 111-61-5 | C20H40O2 | 2193 | 2195-S | 2483 | 53.02±0.00 |
| 112 | Dodecanoic acid | 143-07-7 | C12H24O | 1556 | 1568-S | 2502 | 39.21±25.19 |
| 113 | Furfural | 67-47-0 | C5H4O2 | 828 | 833-S | 2512 | 196.22±95.88 |
| 114 | Diisobutyl phthalate | 84-69-5 | C16H22O | 1857 | 1870-S | 2548 | 310.04±80.46 |
| 115 | Benzoic acid, 2-ethylhexyl ester | 5444-75-7 | C15H22O2 | 1706 | 1735-S | 2598 | 123.94±0.00 |
| 116 | Tetradecanoic acid | 544-63-8 | C14H28O | 1756 | 1768-S | 2685 | 117.65±37.40 |
| 117 | Dibutyl phthalate | 84-74-2 | C16H22O | 1952 | 1965-S | 2705 | 879.15±262.73 |
| 118 | delta-Tetradecalactone | 2721-22-4 | C14H26O | 1918 | 1935-S | 2710 | 55.69±9.27 |
| 119 | Palmitoleic acid | 373-49-9 | C16H30O | 1938 | 1951-S | 2908 | 1484.60±157.00 |
| 120 | n-Hexadecanoic acid | 57-10-3 | C16H32O | 1958 | 1968-S | 2910 | 1269.08±534.40 |
| 121 | 9-Hexadecenoic acid | 2091-29-4 | C16H30O | 1937 | 1942-S | 2957 | 16.98±5.84 |
| 122 | Octadecanoic acid | 57-11-4 | C18H36O2 | 2158 | 2172-S | 3090 | 183.06±0.00 |
| 123 | Oleic Acid | 112-80-1 | C18H34O2 | 2133 | 2141-S | 3172 | 760.27±629.83 |
