# Supplementary material for: Decoding and reconstructing yeast protein flavor based on an integrated sensory-omics approach
Source: Food Chem X. 2026 Jun 9;37:104060. doi: 10.1016/j.fochx.2026.104060 (PMC13293764; doi:10.1016/j.fochx.2026.104060)
Supplement: Supplementary file 3 — Supplementary material 3 [file mmc3.pdf]

Informed Consent Form for Sensory Evaluation

Research Study Title: Decoding and Reconstructing Yeast Protein Flavor Based on an Integrated Sensory-Omics Approach

You are invited to participate in a study of evaluating the aroma profiles and olfactory thresholds of the odorants, which is a scientific research project funded by Beijing Life Science Academy (BLSA) (No. 2023600CA0080), R&D Program of Beijing Municipal Education Commission (No. KM202410011008), and National Natural Science Foundation of China [No. 32102118]. Before you decide whether to participate in this study, please read the following as carefully as possible. It can help you understand the procedure, duration, benefits, risks and discomfort of this study. If you fully understand the instructions and agree to accept the evaluation, please sign at the end of the page for confirmation. If you have any questions, the research director and related staff will explain to you.

Part I: Background and Purpose of This Research

Background: Yeast protein is a highly productive and nutritionally rich microbial protein; however, its development has been limited by its characteristic yeast flavor. To understand the aroma profile of yeast protein and identify its key aroma compounds, we evaluated the aroma characteristics of yeast protein and a recombinant model and determined the threshold levels of the aroma compounds. The results will help elucidate the flavor profile of yeast protein.

Purpose: To identify the key aroma characteristics of yeast protein.

Part II: Study Procedures

Three processes are included in this sensory evaluation.

Panel training. Firstly, panelists were trained by standardized reference solutions representing seven aroma attributes: fatty (E,E)-2,4-decadienal), roasted (2,6-dimethylpyrazine), sour (propanoic acid), sweaty (butanoic acid), green (nonanal), sweety (benzyl alcohol) and almond-like (benzaldehyde) for 3 weeks (twice a week, each training lasting for 1 h). This allowed the panelists to recognize and identify the different aromas present in yeast protein. Secondly, panelists were requested to distinguishthe aromatic characteristics of these compounds on different levels and to be able to correctly rank them according to aroma intensity. This training lasted for 30 min and was performed for 3 times within one week. Finally, twelve panelists will be selected based on the sensitivity and accuracy in aroma compounds recognition.

Quantitative descriptive analysis (QDA). The samples were dispensed into odorless, transparent plastic bottles (which were wrapped in aluminum foil to shield them from state variations), assigned three-digit random codes, and distributed to the evaluators, who rated seven aroma attributes (1-3 points: weak; 4-6 points: moderate; 7-9 points: strong). After completing the evaluation of each set of samples, the evaluators took a 5-minute break. Each sample was tested three times.

Three-alternative forced-choice (3-AFC) procedure. Odorants diluted with water from highest to lowest concentration with a dilution factor of 2. Within each set of three samples, the samples and two blanks were randomly numbered and presented

to panelists. Panelists sniffed three vials with orthonasal perception and were asked to discriminate between different samples. Panelists were allowed to take a 5 min break between the evaluation of different samples to avoid interference.

Part III: Possible Risks and Discomforts

You have been informed that the possible risks and discomforts of this study areas follows: very slight olfactory fatigue. This effect is rare and unlikely to cause you any harm.

Part IV: Offer to Answer Questions and Research Injury Notification

One of the principal investigators or their research associates has offered to and has answered any and all questions regarding your participation in this research study. If you have any further questions or in the event of a research related injury, you can contact the principal investigators.

Part V: Confidentiality of Records

Any information from this study in which you might be identified will be confidential and disclosed only with your permission (by signing this form, however, you allow the study investigators to make you records available). If information generated by this study is published, you will not be identified by name. However, your records may be reviewed to ensure that the study complies with relevant legal and regulatory requirements.

Part VI: Voluntary Participation and Right to Withdraw

Participation in this study is entirely voluntary. You have the right to withdraw your consent and discontinue participation at any time without providing a reason. Withdrawal from the study will not result in any negative consequences.

Part VII: Contact Information

If you have any questions or concerns about the study, you may contact the Principal Investigator, Jiahui Chen, at +86 16636184361 or cjh16636184361@163.com.

Part VIII: Signature for Consent

I have carefully read the information above and understand fully research objectives, processes, risks and my rights as a potential subject in a research experiment involving people as subjects. The investigator has answered all my questions and I agree to be a research subject in this study.

Participant's Signature: [illegible] Date: [illegible]

Participant's Signature: [illegible] Date. [illegible]

Participant's Signature: [illegible] Date: [illegible]

Participant's Signature: [illegible] Date: [illegible]

Participant's Signature: [illegible] Date: [illegible]

Participant's Signature: [illegible] Date: [illegible]

Participant's Signature: [illegible] Date: [illegible]

Participant's Signature: [illegible] Date: [illegible]

Participant's Signature: [illegible] Date. [illegible]

Participant's Signature: [illegible] Date: [illegible]

Participant's Signature: [illegible] Date: [illegible]

Participant's Signature: [illegible] Date. [illegible]

Please print this form for your records.

Thank you for your participation in our study!
