# Supplementary material for: Decoding and reconstructing yeast protein flavor based on an integrated sensory-omics approach
Source: Food Chem X. 2026 Jun 9;37:104060. doi: 10.1016/j.fochx.2026.104060 (PMC13293764; doi:10.1016/j.fochx.2026.104060)
Supplement: Supplementary file 3 [file mmc2.docx]

$$\frac{A}{A_{IS}} = a \times \frac{C}{C_{IS}} + b$$
